# Stable fiber-based polarization-sensitive optical coherence tomography using polarization maintaining common-path interferometer

**DOI:** 10.1117/1.JBO.25.11.116009

**Published:** 2020-11-17

**Authors:** Peijun Tang, Ruikang K. Wang

**Affiliations:** University of Washington, Department of Bioengineering, Seattle, Washington, United States

**Keywords:** optical coherence tomography, polarization, optics fiber, biomedical imaging

## Abstract

**Significance:** Our work advances the development of fiber-based polarization-sensitive optical coherence tomography (PS-OCT) by stabilizing the output polarization state of the light beam when the system is under environmental disturbance. While the fiber-based PS-OCT has been demonstrated previously, it remains a challenge for the traditional fiber-based PS-OCT to obtain a stable measurement when the optic fibers are disturbed by the environment. This important issue is addressed, paving the path for clinical translation of PS-OCT, which can provide a unique perspective of the biological samples.

**Aim:** Polarization maintaining common-path (CP) interferometer is fabricated with the goal of providing a stable fiber-based PS-OCT imaging system that is only responsive to the polarization changes generated by the sample, immune to environmental conditions.

**Approach:** The system is implemented by incorporating a CP interferometer together with polarization maintaining (PM) fibers. The PM fibers are used to preserve the two orthogonal linearly polarized components of the light during propagation. By sharing the CP in the sample and reference arms, any variations in phase retardation can be eliminated between the two channels in the PM fibers. The combination of the PM fiber and the CP interferometer ensures the stability of the output polarization state.

**Results:** The stability of the proposed PS-OCT system is tested when a periodically stressed disturbance is applied to the fibers within the system. Stable *in vivo* PS-OCT images of the mouse thigh are demonstrated.

**Conclusions:** We have demonstrated a stable fiber-based PS-OCT system that combined the PM fiber and the CP configuration together. We have shown that the output polarization states and the system sensitivity can keep stable over time under the environmental disturbances to the system.

## 1 Introduction

Optical coherence tomography (OCT) is a noninvasive optical imaging technique that can generate cross-sectional images of the turbid samples, enabling the visualization of 3D microanatomy with high resolution (1 to 20  μm).^1^ To provide additional contrast when imaging birefringent sample, polarization-sensitive OCT (PS-OCT) has been developed to characterize anisotropic biological structures, such as tendon, muscle, collagen, and nerve fiber bundles.^2^^–^^11^ The initial PS-OCT was based on free-space setup since the bulk air-spaced optical components permit precise control over the polarization state of light in the sample and reference arms.^2^^–^^4^^,^^10^^–^^12^ Compared with the free-space setup, fiber-based interferometers offer distinct advantages in terms of system alignment and handling, desirable for clinical translations. However, because the optical fiber can introduce complex changes in the polarization state of the light, the fiber-based implementation of the PS-OCT is still practically challenging. In the previous studies, polarization-maintaining (PM) fiber that supports the propagation of two orthogonal polarization modes with high isolation was utilized to develop the fiber-based PS-OCT.^5^^–^^9^^,^^13^ Although the two polarization modes of the incident light can be preserved in the PM fiber during their propagation, the phase delay between them is difficult to control since multiple factors, e.g., fiber length, environmental temperature, and strain variations, can cause unpredictable changes in phase. Any disturbance in the PM fiber can induce a change in the output polarization state. This challenge generates a strict requirement for the stability of the system’s working environment, thus difficult to achieve in practical applications, especially in clinical translations.

To mitigate this situation, we propose a facile and stable system that is designed to minimize the changes in the polarization states induced by the optical fibers. In this system, a common-path (CP) interferometer implemented by the PM fibers is first introduced to constitute the fiber-based PS-OCT imaging system. The PM fibers are used to ensure the intensity stability at each polarization detection channel. The CP interferometer, in which the sample and reference beams share the same optical path, helps remove the phase noise^14^ and compensate for additional phase retardation-induced external disturbances. Although CP interferometer has been reported in the free-space PS-OCT system,^12^ its benefits are yet to be demonstrated in the fiber-based PS-OCT system, particularly under the real-world environment, where polarization states within the transmitting fiber are often disturbed. In this paper, the stability of the PS-OCT systems with and without using the PM-CP interferometer is tested in a disturbing environment, where a periodically stressed disturbance is applied to the PM fiber. Stable *in vivo* PS-OCT images of the mouse thigh are demonstrated in the disturbing environment.

## 2 Methods

**Fig. 1:**
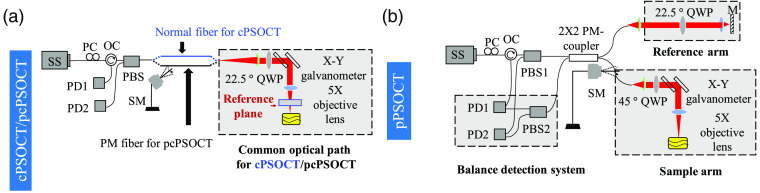
Schematic of the swept-source OCT systems configured to perform (a) typical cPS-OCT and proposed stable pcPS-OCT and (b) traditional pPS-OCT. The reference arm in (a) was constructed by inserting a cover glass plate between the telecentric scan lens and sample. The reference plane was the sample-side optical surface of the reference plate. OC: optical circulator; PBS: polarization beam splitter; PM-coupler: polarization maintaining coupler; QWP: quarter-wave plate; PD: photo detector; PMF: polarization maintaining fiber; SM: stepping motor.

Schematic setup for a common-path PS-OCT (cPS-OCT) system using a normal fiber, a traditional polarization maintaining PS-OCT (pPS-OCT) system, and a stable polarization maintaining CP fiber-based PS-OCT (pcPS-OCT) system is shown in Fig. 1. The light source used in all the systems was a 100-kHz MEMS-VCSEL swept laser source (SL1310V1-20048, Thorlabs), providing a central wavelength of 1310 nm and a spectral tuning range of 100 nm. The laser had an averaged output power of 25 mW. The output of the swept source was sent to a polarization controller and became linearly polarized (LP) through a polarization beam splitter (PBS) (PFC1310A, Thorlabs) before illuminating each PS-OCT system. In the cPS-OCT setup [Fig. 1(a)], the output LP light was sent through a standard telecommunication single-mode fiber (indicated by the blue line) to illuminate the sample, facilitating performance comparison between the cPS-OCT without and with the use of the PM fiber [indicated by the black line shown in Fig. 1(a)], respectively. The CP arm was equipped with a quarter-wave plate (QWP) aligned at 22.5 deg with the LP light (i.e., the light beam before coupled into the normal fiber). Note that in this case, the polarization state of the incident light would be an elliptically polarized light rather than a circularly polarized light since the sample arm and reference arm share the same optical path. When using the single input Jones matrix-based algorithm,^4^ the circularly polarized incident light enables retardation measurements independent of the sample’s axis orientation^15^ while the retardation would not be decoupled from the axis orientation if using the elliptically polarized light as the incident light. Hence, we only provide the polarization state results whose measurements are independent of the input polarization state of the light to demonstrate the stability of the proposed configuration in this work. To extract the phase retardation and the axis orientation information using the proposed stable fiber-based PS-OCT system, two measurements with the QWP aligned at 22.5 deg and −22.5  deg, respectively, can be conducted. Two distinct input polarization states (i.e., two elliptically polarized lights) that illuminating the sample can be achieved with the QWP aligned at these two different angles. When the QWP is aligned at both 22.5 deg and −22.5  deg, the reference signal can be coupled equally into the vertical and horizontal channels. Different from the situation when the QWP aligned at 22.5 deg, a phase delay pi should be added into the complex signal in the horizontal channel digitally to compensate the phase delay introduced by the reference beam. With two distinct input polarization states, the local phase retardation and the local axis orientation can be calculated by the algorithm provided in previous work.^11^

A cover glass plate (BS-50P, Amscope) was inserted between the telecentric scan lens and sample, which was used as the reference plane. The lights coming back from the sample and reference plane were recombined and sent to the PBS, where the interference light was split into horizontal and vertical components and detected by two photodetectors, respectively. In the pcPS-OCT system [Fig. 1(a)], the output LP light is directly sent to the CP arm through the PBS. The LP input ensures the stability of the input polarization state of the light before illuminating the sample. The CP arm and the detection system are the same as the cPS-OCT system. In the pPS-OCT system [Fig. 1(b)], the output LP light was split into the reference arm and the sample arm through a PM coupler (PN1310R5A2, Thorlabs) at a split ratio of 50:50. The reference arm was installed with a QWP with its axis aligned at 22.5 deg with reference to the input polarization state, ensuring that the reflected light was coupled equally into the vertical and horizontal channels. The sample arm was equipped with a QWP aligned at 45 deg with the input polarization state, which makes the LP light to become a circularly polarized light before illuminating the sample. The lights coming back from both the reference and sample arms were recombined and sent to PBS1 (PFC1310A, Thorlabs) and PBS2 (PFC1310A, Thorlabs), respectively. Balanced detection with signal amplification was achieved for both vertical and horizontal channels to collect the interference signals.

The interference signals of all the three PS-OCT setups, recorded by the photodetectors, can be written as: $A_{H,V}(z)=\frac{\sqrt{R_{H,V}(z)}}{2\sqrt{2}}\;\cos[\phi_{H,V}(z)]$, where the subscript variables H and V correspond to signals in the horizontal and vertical channels, respectively. $R_{H,V}(z)$ and $\phi_{H,V}(z)$ are the reflectance of the sample and the OCT signal phase at depth z, respectively.

As mentioned above, the environmental disturbance to the fiber (including both the normal and PM fibers) can induce changes in the output polarization state. The derivations of the generation and compensation of the changes are discussed below. For the PS-OCT measurement, the output polarization state can be determined by three measurands, i.e., the intensities $I_{H}=\langle {A_{H}}^{2}\rangle$ and $I_{V}=\langle {A_{V}}^{2}\rangle$ acquired from each channel and the difference between the phase signals in the two channels: $\phi_{d}(z)=\phi_{H}(z)-\phi_{V}(z)$.^2^ In the cPS-OCT mode, the output polarization state can be affected severely due to the asymmetric design of the normal fiber when a disturbance is applied to it. The end effect is that the intensities in the two orthogonal channels are redistributed and the additional phase difference (PD) is added. Thus, the polarization state of the light beam would not be preserved. For the pPS-OCT mode, the use of the PMF can ensure the stability of the intensity in each channel (i.e., $I_{H}$ and $I_{V}$). However, it does not maintain the PD $\phi_{d}(z)$ between the two channels. When a disturbance is applied to the PM fiber in the sample arm or reference arm, a phase shift $\phi_{\mathrm{He}}$, $\phi_{\mathrm{Ve}}$ induced by the environment can be introduced to the interference signal at each channel. Under this condition, the phase signal can be written as: $\phi_{H,V}(z)=\phi_{\mathrm{Hs},\mathrm{Vs}}(z)+\phi_{\mathrm{He},\mathrm{Ve}}(z)$, where $\phi_{\mathrm{Hs}}\;$ and $\phi_{\mathrm{Vs}}$ are the phase shifts of interest that are induced by the sample. Then, the PD between these two channels can be obtained:

$$\phi_{d}(z)=\phi_{H}(z)\text{-}\phi_{V}(z)=\phi_{\mathrm{ds}}(z)+\phi_{\mathrm{de}}(z),$$ (1)

where $\phi_{\mathrm{ds}}(z)=\phi_{\mathrm{Hs}}(z)\text{-}\phi_{\mathrm{Vs}}(z)$, $\phi_{\mathrm{de}}(z)=\phi_{\mathrm{He}}(z)\text{-}\phi_{\mathrm{Ve}}(z)$. As a consequence, the PD between the two orthogonal polarized components in the traditional pPS-OCT is dependent on both the birefringent property of the sample and the environment disturbance to the PMF, indicating that the output polarization state is sensitive to the environment, even with the use of the PM fibers. For the pcPS-OCT, the reference light is provided by an optical surface of a glass plate proximal to the sample. The reference and sample light beams share a common light path. Because of the CP, the sample and reference light beams would both experience equal phase shifts. Hence, the environment-induced phase shift $\phi_{\mathrm{He},\mathrm{Ve}}$ can be canceled when evaluating the PD between the two channels: $\phi_{\mathrm{de}}(z)=\phi_{\mathrm{He}}(z)-\phi_{\mathrm{Ve}}(z)=0$. Consequently, the PD is obtained as

$$\phi_{d}(z)=\phi_{H}(z)\text{-}\phi_{V}(z)=\phi_{\mathrm{ds}}(z)\text{-}\phi_{\mathrm{de}}(z)=\phi_{\mathrm{ds}}(z),$$ (2)

where the PD is only sensitive to the sample. With the use of the PM fibers, $I_{H}$ and $I_{V}$ would be stable. Therefore, the combination of the PM fibers and the CP configuration makes the output polarization state stable over time even when the system is under a disturbed environment.

## 3 Results

**Fig. 2:**
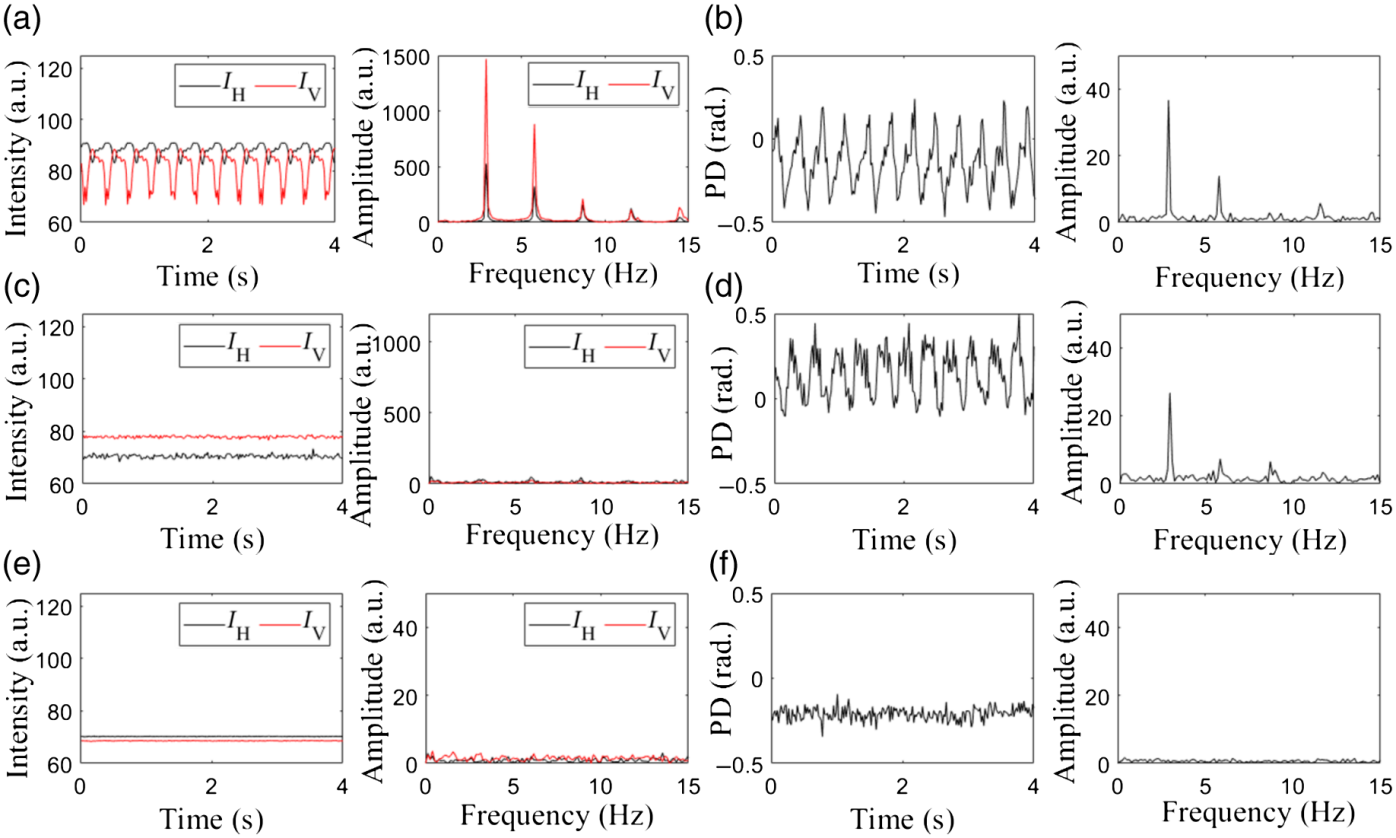
Stability of the PS-OCT signals and the corresponding frequency analyses for three different PS-OCT imaging modes, respectively: (a) and (b) the cPS-OCT without using the PMF; (c) and (d) the traditional pPS-OCT; (e) and (f) the stable pcPS-OCT. For each imaging mode, time course curves of the intensity $I_{H}$ (black curve), $I_{V}$ (red curve), and the PD $\phi_{d}(z)$ over a period of 4 s and their corresponding frequency analyses were obtained by imaging a birefringent plastic cap and plotted as shown.

The necessity of using the above strategy for the stable fiber-based PS-OCT system is revealed by an experimental comparison. A highly birefringent sample (a plastic cap) was imaged by the PS-OCT system in three different setups (Fig. 1). Repeated B-scans were performed to image the plastic cap at 200 fps. The 800 B-scans were acquired within 4 s. During this process, a stick connected to an SM was rotated at 3 Hz to disturb the optic fiber in the sample arm (illustrated in Fig. 1). Then, the $I_{H}$ and $I_{V}$, and $\phi_{d}(z)$ during the periodic disturbance over time were measured. Finally, we calculated the average intensity signal and the average PD signal from a selected region (30×30  pixels) of the sample. The results are provided in Fig. 2.

With the cPS-OCT mode, the time course curves of the average intensity signals acquired from channel 1 ($I_{H}$) and channel 2 ($I_{V}$), respectively, and the corresponding frequency spectra are shown in Fig. 2(a), where the large fluctuations of the intensity signals $I_{H}$ and $I_{V}$ are seen over time. The opposite change trends of the two intensity signals indicate that the change in the birefringent property of the normal fiber can cause a redistribution of the intensities in the two orthogonal polarized channels. Figure 2(b) shows the time course curve of the average PD signal and its frequency spectrum, where the large fluctuations over time are evident. Frequency analyses [Figs. 2(a) and 2(b)] resulted in a main frequency peak at 3 Hz (and its corresponding harmonic signals) in all the signals, indicating $I_{H}$, $I_{V}$, and $\phi_{d}(z)$ were actively modulated by the periodic disturbance of the rotating stick applied to the optic fiber. Thus, such kind of cPS-OCT setup utilizing the common single-mode fibers is highly environmentally sensitive, not practical for clinical applications.

With the PM fibers and the Michelson interferometer, i.e., the pPS-OCT mode, the time course curves of the average $I_{H}$, $I_{V}$, and $\phi_{d}(z)$, and the corresponding frequency spectra are shown in Figs. 2(c) and 2(d), respectively. Because the sample arm and the reference arm were separate, an additional phase shift induced by any environment disturbance in the sample arm led to a vertical translation of the sample in the acquired images. To eliminate this effect, we aligned all the B-scan images before we plot the curves. As expected, the average $I_{H}$ and $I_{V}$ acquired from each channel are seen relatively stable over time [Fig. 2(c)]. The corresponding frequency analysis also indicated that the distribution of the frequency was relatively uniform [see the right plot in Fig. 2(c)]. The results demonstrate that the PM fiber can perverse the two orthogonal polarization states even if there was an environment disturbance to the fibers. However, a periodic 3-Hz fluctuation is persistent in the PD curves [Fig. 2(d)], indicating that the PM fibers cannot protect the stability of the PD between the two orthogonal channels. Consequently, the pPS-OCT system setup would still be prone to environmental disturbance.

Figures 2(e) and 2(f) show the time course curves of average $I_{H}$, $I_{V}$, $\phi_{d}(z)$, and their corresponding frequency spectra, respectively, delivered by employing the PM fibers and the CP interferometer, i.e., pcPS-OCT. We evaluated the signal fluctuation levels for the three curves, resulting in an STD value of 0.05, 0.06, and 0.03 for $I_{H}$, $I_{V}$, and $\phi_{d}(z)$, respectively. The results indicate that the pcPS-OCT provides not only the stable $I_{H}$ and $I_{V}$ in channel 1 and channel 2 but also the stable PD $\phi_{d}(z)$ under the disturbing environment over time. The corresponding frequency analyses confirm this conclusion [see the right plots in Figs. 2(e) and 2(f)]. This is because the PMF preserves the stability of the intensity signals from each channel, and the CP configuration enables automatic compensation of the phase shifts induced by any environmental disturbances.

**Fig. 3:**
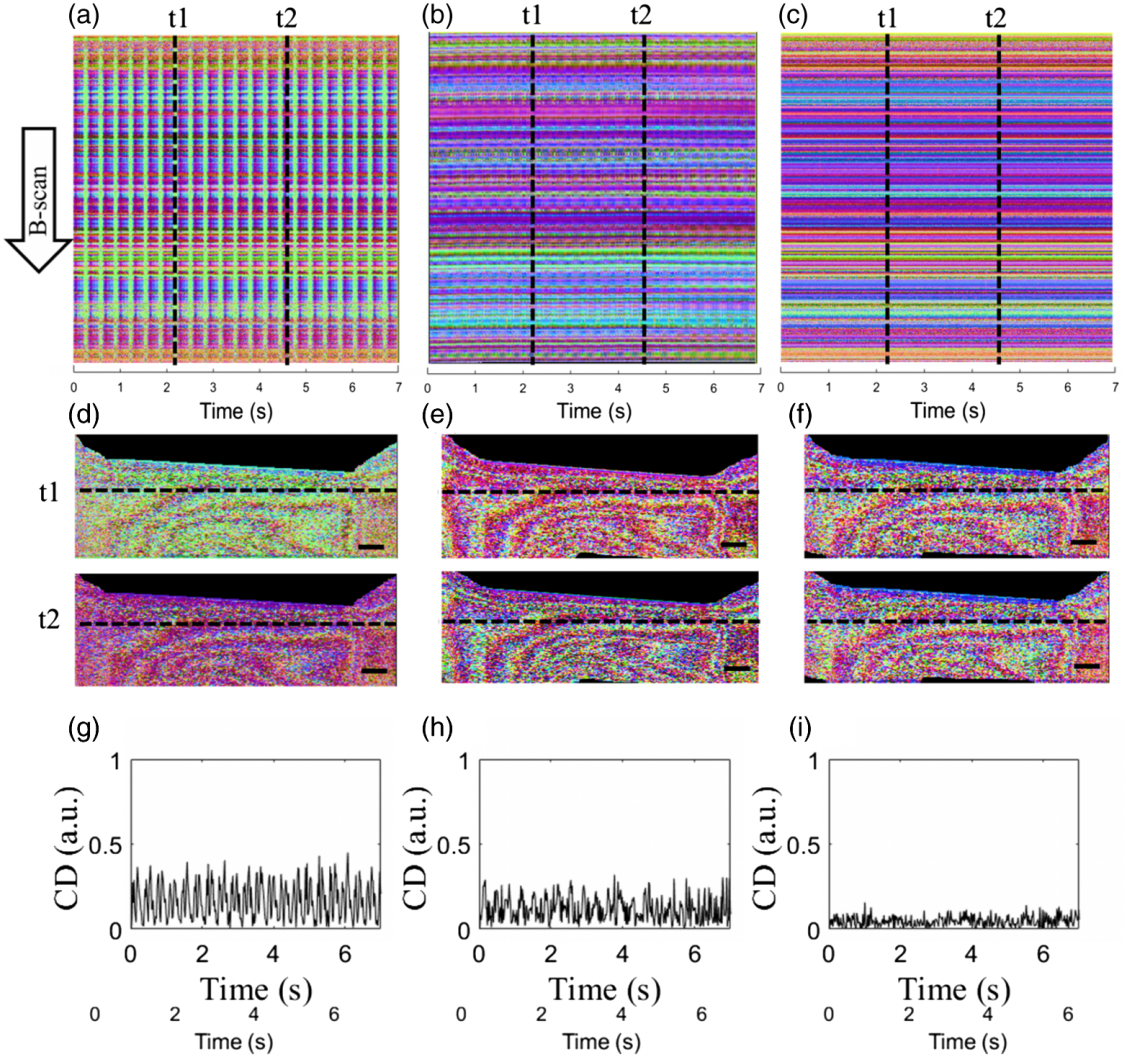
Stability of the polarization state images and the corresponding CD curves of a plastic cap obtained by three PS-OCT imaging modes: (left column) the cPS-OCT without using PMF; (middle column) the pPS-OCT; and (right column) the stable pcPS-OCT. (a)–(c) The polarization state images sliced at the depth of 500  μm of the 3D time-lapse repeated B-scan dataset are shown; (d)–(f) the corresponding B-scan cross-sectional polarization state images at the time instants of $t_{1}=2.3\;s$ and $t_{2}=4.7\;s$, respectively, are shown; (g)–(h) the averaged CD signals calculated from (a)–(c), respectively, are shown. Scale bar=500  μm.

To visually assess the stability of the output polarization state overtime, time-lapsed PS-OCT images (B-scan) of the plastic cap were captured while the system was subject to the constant periodic stress disturbance. To show the results, the output polarization state was reported by the use of the color-encoding scheme, as described in Ref. 10, where three primary colors of red, green, and blue are used to code Stokes parameters of Q, U, and V, respectively, which can be used to represent and visualize each unique polarization state. Figures 3(a)–3(f) show the stability of the polarization state images of the plastic cap over a time period of 7 s captured by the three imaging modes, as stated above. Figures 3(a)–3(c) illustrate time-lapsed PS-OCT images sliced at a fixed depth of 500  μm through the sample, i.e., vertical axis indicates the B-scan at the depth of 500  μm and horizontal axis represents time. Figures 3(d)–3(f) illustrate the B-scan PS-OCT images at two instants of $t_{1}=2.3\;s$ and $t_{2}=4.7\;s$ [at the positions indicated by dashed lines in Figs. 3(a)–3(c), respectively] to indicate how stable the B-scan images are. In these cross-sectional B-scan images, typical band patterns induced by the birefringent property of the plastic cap can be seen. The inhomogeneous banded patterns in the cumulative polarization state images suggest a varying birefringent property in the plastic cap. Because each color represents a unique polarization state, we utilized the color difference (CD) to quantitatively describe the stability of the polarization state by the CD equation:

$$\mathrm{CD}(t)=\sqrt{{\left[R(t)-R(t_{0})\right]}^{2}+{\left[G(t)-G(t_{0})\right]}^{2}+{\left[B(t)-B(t_{0})\right]}^{2}},$$ (3)

where R(t), G(t), and B(t) are the red, green, and blue values of one pixel at time t, respectively, $t_{0}$ is the start time. Figures 3(g)–3(i) show the average CD signals calculated from Figs. 3(a)–3(c), respectively. The CD signals vary relatively dramatic overtime in Figs. 3(g) and 3(h) with an STD value of 0.11 and 0.07, respectively, whereas it is relatively constant with an STD value of 0.02 in Fig. 3(i). It is clear that the pcPS-OCT imaging mode provides constant and stable imaging results over time despite the system was subject to an environmental distribution, which is not the case for cPS-OCT and pPS-OCT setups.

**Fig. 4:**
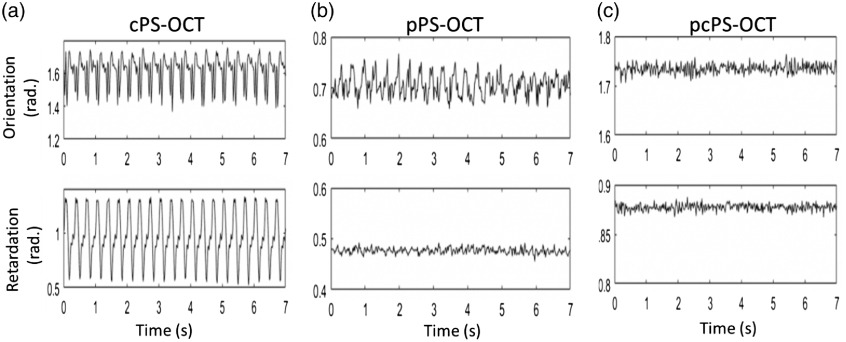
Stability of (a) the cPS-OCT without using PM fibers; (b) the pPS-OCT; and (c) the stable pcPS-OCT as assessed by the measurements of axis orientation (top) and phase retardation (bottom), respectively.

The stability over time was also assessed by the corresponding measurements of axis orientation and phase retardation for the three setups (Fig. 4). The average orientation signals related to PD for cPS-OCT [Fig. 4(a)] and pPS-OCT [Fig. 4(b)] vary relatively dramatically over time with an STD value of 0.982 and 0.873, respectively, however, it is relatively constant with an STD value of 0.009 in pcPS-OCT [Fig. 4(c)]. This result shows the necessity of the use of the CP for the orientation measurement in the PM fiber-based PS-OCT because the orientation is highly sensitive to the PD. The average phase retardation signals related to intensity signals in Fig. 4(a) show a large periodic fluctuation over time with an STD value of 0.231, however, it is relatively constant with an STD value of 0.007 and 0.003 in Figs. 4(b) and 4(c), respectively.

**Fig. 5:**
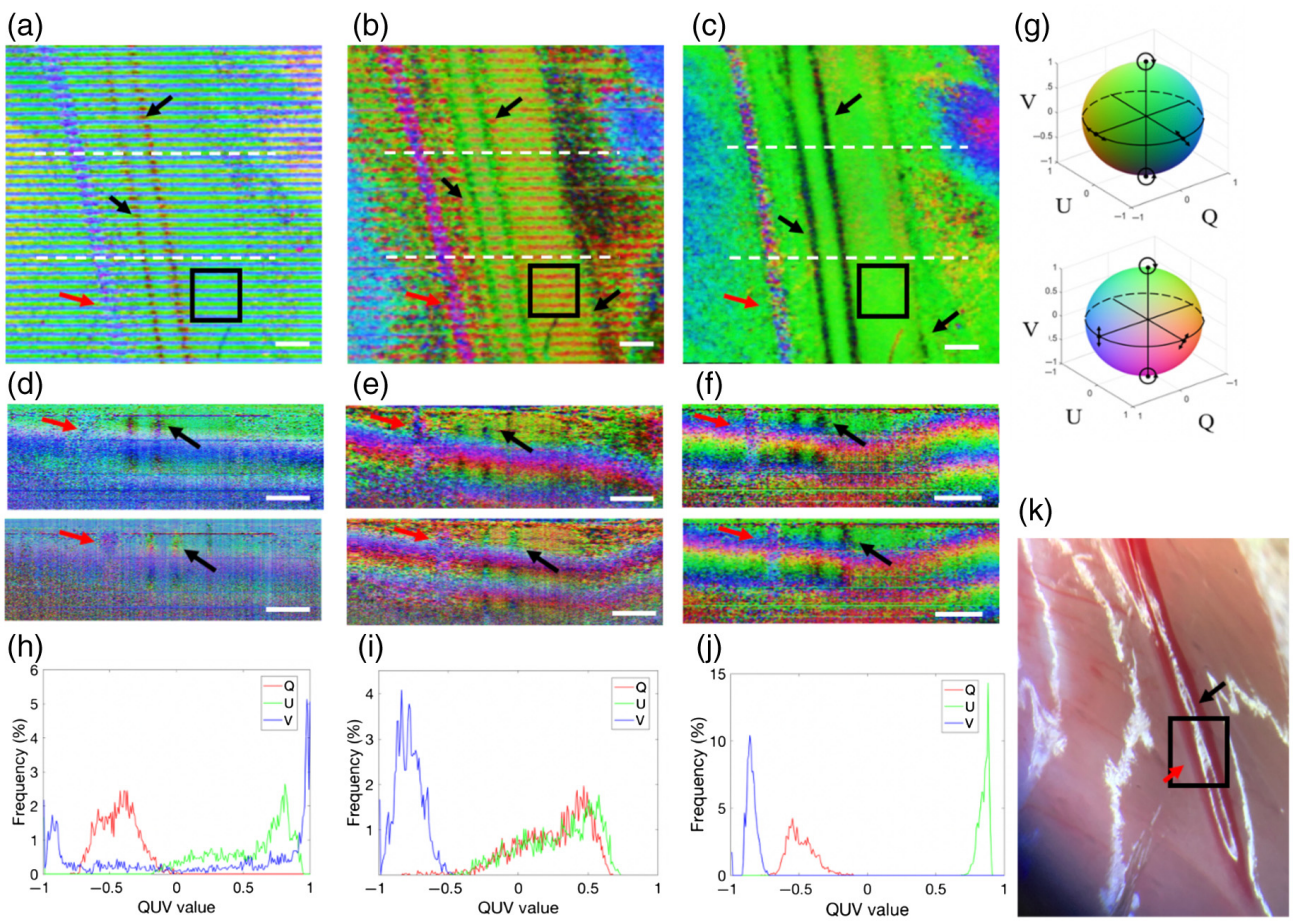
The 3D *in-vivo* polarization state images of the mouse thigh, the corresponding B-scan images, and the histograms of the distributions of QUV values obtained from the region of interest (ROI) by using (a), (d), and (h) the cPS-OCT without using the PM fibers; (b), (e), and (i) the pPS-OCT; (c), (f), and (j) the stable pcPS-OCT. (a)–(c) The color-coded 3D images of the mouse thigh are shown. (d)–(f) The corresponding B-scan images at the positions marked by the dashed lines in the top panel, respectively, are shown. (h)–(i) The histograms of the distributions of QUV values obtained from the ROI indicated by the box in (a)–(c), respectively, are shown. (g) The front side and back side of the color-encoded Poincare sphere. Each color on the surface of the sphere represents a unique polarization state. (k) The photograph of the mouse thigh. Red arrow: nerve bundle; black arrows: vascular walls of artery and veins. The scale bar in (a)–(f) = 500  μm.

To test the performance of the proposed pcPS-OCT system for *in vivo* imaging, 3D PS-OCT images of a mouse thigh were obtained by the three imaging modes under the environmental disturbances (Fig. 5). The 3D scans were performed to image the mouse thigh with a B-scan rate at 200 fps. The A-scans number per B-scan is 800, the B-scans number per C-scan is 2400, which required 12 s to acquire. At each certain location, B-scan was repeated three times and was averaged to remove the speckle noise. All experimental procedures in this study were approved by the Institutional Animal Care and Use Committee (IACUC) of the University of Washington and conducted in accordance with the ARRIVE guidelines. The photograph of the exposed mouse thigh is shown at the top right of Fig. 5(a), where the nerve bundles and the vasculature were indicated by the red and black arrows. Figures 5(a) and 5(b) show the 3D polarization state images of the mouse thigh by using the cPS-OCT without using the PM fibers and the traditional pPS-OCT, respectively. As mentioned above, these two configurations cannot preserve the output polarization states when there is an environmental disturbance applied to the fibers. Although the birefringent components, for example, the nerve bundle and the vascular walls indicated by the red and black arrows can be roughly differentiated, they are disturbed severely by the artifacts induced by the stress disturbance to the fiber, as expected. For comparison, Figs. 5(d) and 5(e) show the corresponding cross-sectional images located at the regions indicated by the white dash lines in Figs. 5(a) and 5(b), respectively, where, due to the artifacts induced by the environmental disturbances, the imaging contrasts of the vessel walls and nerve bundles vary dramatically from one position to another, demonstrating the difficulty to differentiate them in the PS-OCT images. Figure 5(c) shows the 3D image of the mouse thigh with the proposed pcPS-OCT configuration. The image is very stable over time, and periodic artifacts are absent. Figure 5(f) shows the cross-sectional images selected at two locations in Fig. 5(c). The contrast of the nerve bundles and vascular walls keeps almost constant. To quantify the differences in the approaches, we selected a region enclosed by the boxes in Figs. 5(a)–5(c), respectively, to do the statistical analysis. The region was located at the muscle group, which is supposed to have a relatively homogenous birefringent property. The distribution of the pixel number of Q, U, and V within this region was shown in the histograms Figs. 5(h)–5(j), respectively. In Figs. 5(h) and 5(i), the distributions of the pixel number of Q, U, and V are spread out. However, in Fig. 5(j), the distributions of the Q, U, and V are concentrated, demonstrating that the measured output polarization states in this region are relatively consistent over time, which is expected. These results demonstrate the robustness of the proposed pcPS-OCT setup for *in vivo* imaging applications.

## 4 Limitations and Conclusions

While we have demonstrated the usefulness of the proposed stable fiber-based pcPS-OCT system setup, further improvement is required to include treatments that improve the imaging performance of the CP interferometer. By inserting a glass plate near the sample as the reference mirror can potentially lead to several issues for *in vivo* imaging: (1) the intensity of the reference light beam may vary during imaging scanning because the reference beam is scanned with the probing beam and (2) the field-of-view is limited because in the large scanning region, it is difficult to guarantee the stability of the reference beam as the incident angle of the light is large. To mitigate these issues, the reference plane should be set before the galvoscanner to avoid the scanning of the reference beam. This can be achieved by utilizing the fiber end surface (purposely polished if needed) as the surrogate for the reference beam reflector. While such design could impose issues for spectral-domain OCT system implementation, it is feasible for the SS-OCT because the swept-source laser has been demonstrated to provide extremely long coherence length (>200  mm).^16^ In this case, however, there needs additional care of dispersion compensation in the system because there is a huge imbalance of the wavelength dispersion experienced between the reference path and the sample path. In addition, the reference power may vary when the fiber is moved during imaging that may induce a change in the polarization state in the fiber.

In conclusion, we have demonstrated a stable fiber-based PS-OCT system that combined the PM fiber and the CP configuration together. We have shown that the output polarization states and the system sensitivity can keep stable over time under the environmental disturbances to the system. By using this method, we have shown its ability to provide a stable *in vivo* 3D PS-OCT images of the mouse thigh under environmental disturbances. It is hoped that the proposed stable fiber-based PS-OCT system setup would have been useful for practical applications in the investigations of birefringent tissue samples both preclinically and clinically.
